# Social learning is triggered by environmental cues in immigrant birds

**DOI:** 10.1371/journal.pbio.3002904

**Published:** 2024-11-15

**Authors:** Rachel A. Harrison

**Affiliations:** Department of Anthropology, Durham University, Durham, United Kingdom

## Abstract

After dispersal, what cues trigger social learning in immigrants? This Primer explores a new PLOS Biology study in wild-caught great tits which suggests that changes in the physical environment, rather than the social environment, are key in prompting social learning by immigrants.

## Teaser

After dispersal, what cues trigger social learning in immigrants? A new study in wild-caught great tits in *PLOS Biology* suggests that changes in the physical environment, rather than the social environment, are key in prompting social learning by immigrants.

Social learning is learning via observation of, or interaction with, another animal [1]. This definition is broad, and as such social learning can include both the acquisition of novel behaviours to be used over the long-term, such as chimpanzees learning to use moss as a sponge to drink water [2], and the short-term influence of social information on an individual’s behaviour [3], such as bumblebees feeding from the same flower as conspecifics [4]. Social learning is a powerful way for individuals to acquire adaptive information or behaviours without paying the cost of potentially risky and time-consuming individual exploration or trial and error learning. However, while social learning is widespread in the animal kingdom, theoretical models predict that in order to be adaptive it should not be used indiscriminately, but rather that individuals will follow “social learning strategies” dictating when, what, and from whom they learn [5].

Models also suggest that immigrants should be particularly likely to learn socially [6], an argument that makes intuitive sense as immigrants may find themselves in novel environments in which their existing behavioural repertoire is suboptimal, and they have access to new group mates who have more experience of this environment and are in possession of a behavioural repertoire more suited to it. This conjecture is also supported by observational data across a range of species. These studies have often relied on observations of naturally occurring dispersal events and have tended to show that immigrants adopt the preferences of their new group [7,8]. However, these studies generally have not been able to disentangle the influence of a change in the physical environment versus the social environment—as immigrants move into new groups, they experience a shift in both, and therefore it has not been possible to examine which has the larger influence on their behaviour.

Chimento and colleagues’ new study in *PLOS Biology* [9] employs an elegant design to address this question. Wild-caught great tits were kept in small groups in aviaries mimicking either pine or deciduous forest and provided with automated puzzle boxes with 2 potential solutions. A “tutor” individual was trained to perform one solution in each group, and this behaviour then spread so that groups had distinct solution preferences. Immigration events were then simulated by moving individuals between these established groups, and the impact of environmental change was explored by moving individuals into groups in which the aviary habitat and/or the payoff offered by the puzzle box (with the alternative solution to the immigrants’ trained solution providing a higher payoff) could differ from their source group. According to the experimental design, either both, one or neither of these environmental factors differed from their source group. Meanwhile, in groups receiving immigrant birds, residents experienced a change in their social environment, but no change in the physical environment.

**Fig 1:**
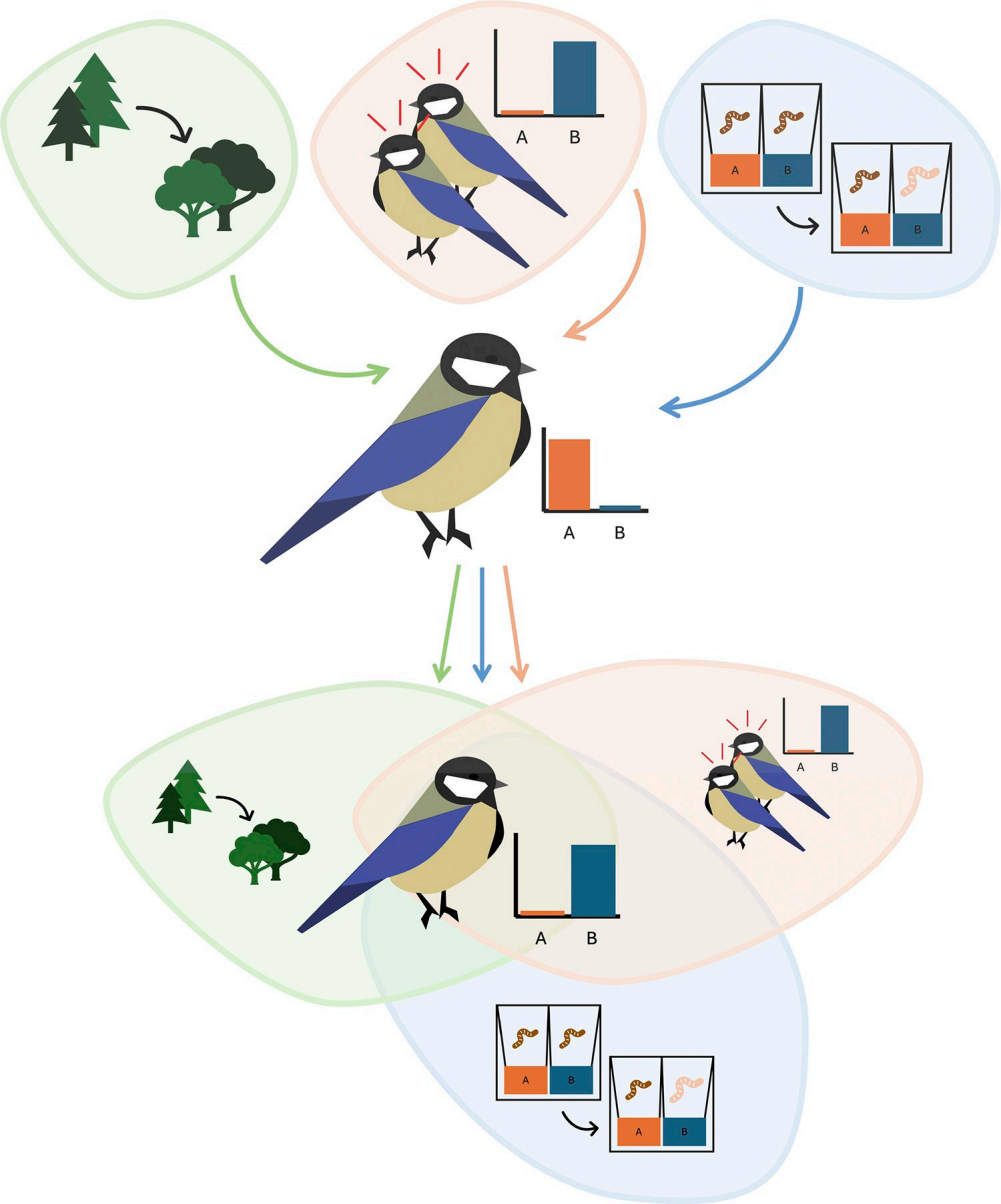
The influence of the physical environment, payoff structure, and social environment on great tit social learning. Chimento and colleagues [9] investigated how different environmental cues influenced social learning in wild-caught great tits. Having established a preference for one solution to an artificial foraging task, birds were moved between aviaries in simulated immigration events. When this move was to an aviary in which the physical environment (pine versus deciduous vegetation) differed from their home aviary and the payoff structure of the task differed such that an alternative solution was more rewarding, immigrants followed payoff-biased social learning and adopted the alternative solution preferred by their new group. Exposure to new group mates performing an alternative solution was not sufficient to trigger social learning if the physical environment and payoff structure matched their home aviary.

The study found that immigrant great tits used social information when both the physical environment and the payoff structure changed (Fig 1), while when only the payoff structure changed, they appeared to rely more-so on individual learning to gradually acquire the more rewarding behaviour. When neither the physical environment nor payoff structure changed, immigrants’ exploration of the alternative solution modelled by their group was limited. Meanwhile, residents in the same experiment experienced a change in social environment (the arrival of new immigrant group members), and did not alter their behaviour, beyond initial sampling the immigrant behaviour when payoffs were symmetrical.

Social learning in immigrants thus appears to be flexible and is triggered by a change in the physical environment, rather than being due to an underlying life-history state—Chimento and colleagues found that it was not a response to the act of dispersal alone, but contingent upon the immigrant’s environment after dispersal. Residents and immigrants who did not experience a shift in payoff structure were subject to some social influence immediately following the immigration event, evidenced by their exploration of the alternative solution demonstrated by their new group mates. However, this change in social environment alone did not trigger a shift in preferences of the type observed in previous observational studies in which changes in the physical and social environment were conflated [7,8]. This finding offers a new perspective when interpreting existing observations of social learning following migration and dispersal, particularly in species such as great apes in which controlled experiments of this type would be impractical or unethical. It has been argued that social learning in immigrants may primarily serve to aid in social integration [10], a stance called into question by Chimento and colleagues’ finding that a change in social environment alone had little influence on great tits’ behaviour. However, the extent to which this finding can be generalised to species with different social structures and in which behaviour may serve as a more potent indicator of group membership has yet to be seen. An additional area for further research is the question of whether this finding applies only to social influence on the production of behaviour, or whether spatial variability is also a key factor in triggering the acquisition of novel behaviour [3].
